# Residential mobility predicts COVID-19 and seasonal flu vaccination behaviors in the United States

**DOI:** 10.3389/fpubh.2022.1064962

**Published:** 2023-01-27

**Authors:** Ning Zhang, Tao Jiang, Ying Zhang, Gang Zhao

**Affiliations:** ^1^Department of Social Medicine, School of Public Health and Center for Clinical Big Data Statistics, The Second Affiliated Hospital of Zhejiang University School of Medicine, Hangzhou, China; ^2^Institute for Policy Research, Northwestern University, Evanston, IL, United States; ^3^Center for Disease Control and Prevention of Hangzhou, Hangzhou, China

**Keywords:** residential mobility, COVID-19, seasonal flu, vaccination behaviors, public health

## Abstract

**Aim:**

Vaccination is one of the most effective strategies to contain the transmission of infectious diseases; however, people's intentions and behavior for vaccination vary across different regions and countries around the world. It is not clear how socioecological factors such as residential mobility influence people's vaccination behaviors for infectious diseases.

**Methods:**

We analyzed public data on residential mobility and vaccination rates for COVID-19 and seasonal flu in the United States and explored how residential mobility in the previous year influenced vaccination rates for COVID-19 and seasonal flu (2011–2018) across 50 states of the US. The data were accessed and analyzed in 2021.

**Results:**

Study 1 demonstrated that collective-level residential mobility predicted COVID-19 vaccination rates across the United States (*B* = −168.162, 95% CI [−307.097, −29.227], adjusted *R*^2^ = 0.091, *p* = 0.019). Study 2 corroborated this finding by documenting that collective-level residential mobility predicted vaccination rates for seasonal flu from 2011 to 2018 across the United States (*B* = −0.789, 95% CI = [−1.018, −0.56], adjusted *R*^2^ = 0.222, *p* < 0.001). The link between residential mobility and vaccination behavior was robust after controlling relevant variables, including collectivism, cultural tightness–looseness, and sociodemographic variables.

**Conclusions:**

Our research demonstrated that residential mobility is an important socioecological factor that influences people's vaccination behaviors for COVID-19 and seasonal flu. The results enrich our understanding of the socioecological factors that influence vaccination behaviors and have implications for developing tailored interventions to promote vaccination during pandemics of infectious diseases.

## Introduction

Residential mobility, defined as the frequency of moving at the individual level or the percentage of residents in a neighborhood, city, province, or country that moved at the collective level during a certain period, is an increasing trend around the world (1, 2). For example, in 2020, more than 276 million Chinese moved to new provinces for work, education, or family reunion in China, accounting for 26.64% of China's total population (3). According to the American Community Survey, approximately 13% of Americans moved each year during the past 5 years (4). Despite the negative impacts of the COVID-19 pandemic on international travel, the International Organization for Migration (5) reported that there were 281 million international migrants in 2020 around the world. Therefore, researchers are advocating the importance of understanding the psychological consequences of residential mobility on human minds and behavior (1, 2).

Although residential mobility is primarily driven by peoples' desire to pursue better education, work, and happiness (2), it can also increase the risk for transmission of infectious diseases (e.g., incidences of COVID-19 cases) (6). For instance, recent research found that relational mobility—the extent that one can form new relationships or exit current relationships easily in a given society (7)—significantly predicted the spread of COVID-19 across countries during the early stage of the outbreak (8). Research during the Omicron surge in the United States also found that a decline in mobility was significantly associated with decline in incidences of COVID-19 infections between December 2021 and February 2022 (9). Similarly, Guo and colleagues found that vaccination slows the spread of COVID-19 in countries with low mobility more than in those countries with high mobility (10). Social distancing is, accordingly, recommended as one of the most important health-protective behaviors to contain COVID-19 (11–15).

With the COVID-19 pandemic continuing to spread around the world, governments are advocating for eligible residents to be vaccinated for COVID-19. Vaccination is one of the most effective, preventive strategies to reduce the transmission of infectious diseases (16–19); however, many people are hesitant about vaccination (20, 21). As vaccination is a necessary strategy to reach herd immunity, receiving vaccination could be viewed as a prosocial behavior as it not only protects oneself from infection but also contributes to reaching herd immunity, which, in turn, protects both those being vaccinated and other people, especially vulnerable groups (e.g., young children and elders with multiple chronic diseases) who are not eligible for vaccination, from infection. Recent research found that people with a higher level of identification with their community reported a higher level of willingness to engage in community-related prosocial behavior, which, in turn, predicted a higher level of vaccination intention for COVID-19 (22). Similarly, residents with a stronger national identity also reported greater engagement in public health behaviors (e.g., lower mobility, spatial distancing, and stricter hygiene practices) and higher support for public health policies during the COVID-19 pandemic around the world (23).

Pioneering research by Oishi and colleagues on the psychological consequences of residential mobility demonstrated that it is associated with the primacy of personal (vs. collective) self and “duty-free” (vs. obligatory) friendships and group memberships (1, 3, 24, 25). For instance, residential mobility predicted a lower likelihood of engaging in procommunity behaviors (e.g., purchasing a “critical habitat” plate to support the preservation of the environment in their home state) (24). However, it is not clear whether similar effects would be observed for engagement in prosocial health-preventive behaviors (e.g., vaccination). Pandemics of infectious diseases (e.g., COVID-19) pose multilevel social dilemmas between individuals, organizations, communities, and nations around the world (23). For example, individuals need to engage in health-protective behaviors (e.g., wearing face masks and social distancing) to contain the spread of COVID-19, which may be inconvenient and uncomfortable for themselves (23, 26). Multilevel cooperation to prioritize collective welfare over personal convenience is crucial for tackling crises such as the COVID-19 pandemic (23, 27–29). However, the rising trend of residential mobility could be a barrier to increasing compliance and adherence to infection control as it reduces people's likelihood of engaging in community-related prosocial behaviors (2).

Inspired by the research on the psychological consequences of residential mobility, we hypothesized that people from an area with a higher level of residential mobility would be less likely to receive the vaccination. However, no previous research explored how residential mobility influences people's vaccination behavior during pandemics of infectious diseases. The current research filled this gap by investigating how residential mobility at the collective level predicted people's actual vaccination rates for COVID-19 (Study 1) and seasonal influenza (Study 2) in the United States.

## Materials and methods

### Study 1 residential mobility reduces COVID-19 vaccination rates in the United States

#### Measures

##### Residential mobility

We used the American Community Survey (ACS) of 2019 to calculate residential mobility in 50 states of the US (30). Since the state-level residential mobility refers to the percentage of those who move during a certain period in this state, those who reported staying at the same house 1 year ago were seen as non-floating population and those who reported any movement, regardless of movements between states or within states, were seen as floating population. For example, the residential mobility rate of 2019 = (population 1 year and over of a state in 2019—same house 1 year ago)/population 1 year and over of a state in 2019. A higher level of residential mobility indicates that there is a higher percentage of the floating population in that state.

##### COVID-19 vaccination

*Percent of Total Population Fully Vaccinated by State of Residence* and *Percent of Total Population with at least One Dose by State of Residence* were used as two indicators of COVID-19 vaccination rates across the United States (31).

##### COVID-19 severity

Because people in more severely affected regions may have stronger motivation to vaccinate, we controlled for the total case rate per 100,000 people and death rate per 100,000 people (31).

##### Collectivism

We used the state-level collectivism index from Vandello and Cohen (31) as a control variable because collectivism may affect health-protective behaviors during COVID-19 as revealed by Lu and colleagues in their studies in the United States and around the world (26). A higher score indicates a higher level of collectivism. This index is the most widely used indicator of collectivism at the state level within the United States (32).

##### Tightness–looseness

State-level tightness and looseness is a correlated but different variable from collectivism (26, 32). We used state tightness–looseness scores from Harrington and Gelfand (32) as a control variable because greater state tightness scores indicated stronger enforced rules and less tolerance for deviance and, thus, may affect vaccination behavior for COVID-19.

##### Social economic and demographics

Previous studies showed that socioeconomic statuses such as educational level and income affected COVID-19 vaccination intention (33, 34). Therefore, we controlled socioeconomic and demographic variables at the state level, including the educational level of 2015–2019, the per-capita income of 2019, the poverty rate of 2019, and the real GDP in chained dollars of 2019. The socioeconomic and demographic data were quoted from US Economic Research Service (ERS) (35). The ERS State Fact Sheets provide information on population, income, poverty, education, and other key information of each state of the United States. The real GDP in chained dollars of each state in 2019 was taken from the Bureau of Economic Analysis (BEA) as a more stable indicator of economic conditions by removing the effects of price changes (36).

### Study 2 residential mobility reduces seasonal flu vaccination rates across the United States

#### Measures

##### Residential mobility

We used the American Community Survey (ACS) from 2011 to 2018 to calculate the residential mobility of 50 states in the US. The residential mobility rate was calculated using the same method as in Study 1 (31).

##### Seasonal flu vaccination coverage

Seasonal influenza vaccination coverage was quoted from the Centers for Disease Control and Prevention (37). We used the end-of-season flu vaccination coverage from 2011–2012 flu season to 2018–2019 flu season. To be specific, for example, the flu vaccination coverage of the 2018–2019 flu season was the cumulative percentage in May 2019. The flu vaccination coverage estimates for the 2013–2014 flu season in California and Mississippi and the 2018–2019 flu season in New Jersey were missing. Therefore, a total of 397 matched data for flu vaccination rates were included in the final analysis.

##### Controlled variables

Similar to Study 1, we controlled the *state-level collectivism index* from Vandello and Cohen (38) and the *state-level tightness–looseness scores* from Harrington and Gelfand (32). We also controlled *real per-capita personal income* (chained 2012 dollars) from 2011 to 2018, quoted from the Bureau of Economic Analysis (BEA) (39), and the *real GDP in chained dollars* from 2011 to 2018, taken from the Bureau of Economic Analysis (BEA) (40). As the college completion rate is not available for every year between 2011 and 2018, it is not included as a covariate in this study.

### Statistical analyses

All data analyzes were conducted using IBM SPSS Statistics for Windows (41). Data for both studies are available from the website as referenced in the Methods section. The data were accessed and analyzed in 2021.

This study did not require IRB approval because it involved analyzes of a publicly available, fully de-identified dataset.

## Results

### Study 1

Descriptive statistics and bivariate correlations are displayed in Table 1. Percentage of the total number of population fully vaccinated for COVID-19 was negatively correlated with residential mobility (*r* = −0.384, *p* = 0.006), tightness (*r* = −0.745, *p* < 0.001), total case rate per 100,000 (*r* = −0.489, *p* < 0.001), and poverty rate (*r* = −0.573, *p* < 0.001) and positively correlated with per-capita income (*r* = 0.651, *p* < 0.001), college completion rate (*r* = 0.778, *p* < 0.001). Percentage of total population with at least one-dose COVID-19 vaccinations were negatively correlated with residential mobility (*r* = −0.331, *p* = 0.019), tightness (*r* = −0.706, *p* < 0.001), total case rate per 100,000 (*r* = −0.473, *p* < 0.001), poverty rate (*r* = −0.541, *p* < 0.001) and positively correlated with per-capita income (*r* = 0.578, *p* < 0.001), college completion rate (*r* = 0.686, *p* < 0.001).

**Table 1:** Descriptive statistics and bivariate correlations of main variables.

	**M**	**SD**	**1**	**2**	**3**	**4**	**5**	**6**	**7**	**8**	**8**	**10**
1. Residential mobility	0.141	0.019										
2. Collectivism	50.080	11.337	−0.455^**^									
3. Tightness	50.139	12.596	0.071	0.228								
4. Case Rate per 100,000	10,220.140	2,436.400	0.142	−0.145	0.441^**^							
5. Death rate per 100,000	167.580	60.702	−0.328^*^	0.124	0.365^**^	0.653^**^						
6. Per–capita income	54,235.700	8,631.768	−0.201	−0.111	−0.633^**^	−0.196	−0.037					
7. Poverty rate (percent)	12.190	2.650	−0.120	0.253	0.647^**^	0.241	0.424^**^	−0.692^**^				
8. Completing college (percent)	31.238	5.234	−0.170	−0.112	−0.602^**^	−0.315^*^	−0.169	0.809^**^	−0.752^**^			
9. Real GDP in chained dollars	376,087.134	492,091.100	−0.243	0.213	−0.153	−0.027	0.118	0.336^*^	0.007	0.214		
10. Total Pop Fully Vaccinated (percent)	47.466	8.492	−0.384^**^	−0.136	−0.745^**^	−0.489^**^	−0.213	0.651^**^	−0.573^**^	0.778^**^	0.106	
11.Total Pop with at least One Dose (percent)	54.004	9.528	−0.331^*^	−0.112	−0.706^**^	−0.473^**^	−0.209	0.578^**^	−0.541^**^	0.686^**^	0.117	0.919^**^

* *p* < 0.05,

** *p* < 0.01.

Regression analysis showed that state-level residential mobility predicted the percentage of the total population fully vaccinated for COVID-19 rate across each state (*B* = −173.45, *SE* = 60.3, *t* = −2.877, *p* = 0.006, 95% CI [−294.66, −52.242], see Model 1 of Table 2), and the regression model is significant (adjusted *R*^2^ = 0.129, *p* = 0.006, see Model 1 of **Table 4**), such that states with a higher level of residential mobility had a lower percentage of people fully vaccinated for COVID-19. State-level residential mobility remained as a significant predictor of the percentage of the total population fully vaccinated with COVID-19 vaccinations after controlling for collectivism, tightness–looseness, total case rate per 100,000, death rate per 100,000, per-capita income, poverty rate, completing college rate, and real GDP in chained dollars (*B* = −153.037, *SE* = 37.099, *t* = −4.125, *p* < 0.001, 95% CI [-228.017,−78.057], see Model 2 of Table 2), and the regression model is significant (adjusted *R*^2^ = 0.821, *p* < 0.001, see Model 2 of **Table 4**). The results also revealed that states with a higher level of collectivism, cultural tightness, and a higher level of college completion rate had a higher level of COVID-19 vaccination rates (see Table 2).

**Table 2:** Predictors of total population fully vaccinated for COVID-19 across U.S.

		***B***	***SE***	***Beta***	***t***	***p***
Model 1	1. Residential mobility	−173.45	60.3	−0.384	−2.877	0.006
Model 2	1. Residential mobility	−153.04	37.1	−0.338	−4.125	0.000
	2. Collectivism	−0.128	0.057	−0.170	−2.255	0.030
	3. Tightness	−0.272	0.065	−0.404	−4.208	0.000
	4. Case Rate per 100 k	−0.001	0.000	−0.157	−1.459	0.152
	5. Death Rate per 100 k	0.002	0.016	0.011	0.094	0.925
	6. Per-capita income	−0.000	0.000	−0.085	−0.675	0.503
	7. Poverty rate (percent)	0.323	0.434	0.101	0.744	0.461
	8. Completing college (percent)	0.938	0.202	0.578	4.641	0.000
	9. Real GDP in chained dollars	−1.775	0.000	−0.103	−1.437	0.159

State-level residential mobility also predicts the percentage of the total population with at least one-dose vaccination for COVID-19 across each state (*B* = −168.162, *SE* = 69.1, *t* = −2.434, *p* = 0.019, 95% CI [-307.097, −29.227], see Model 3 of Table 3). The regression model is significant (adjusted *R*^2^ = 0.091, *p* = 0.019, see Model 3 of Table 4). State-level residential mobility remained as a significant predictor of the percentage of the total population with at least one-dose vaccination for COVID-19 after controlling for collectivism, tightness–looseness, total case rate per 100,000, death rate per 100,000, per-capita income, poverty rate, completing college rate, and real GDP in chained dollars (*B* = −137.383, *SE* = 59.211, *t* = −2.32, *p* = 0.026, 95%CI [−257.054, −17.712], see Model 4 of Table 3), and the regression model is significant (adjusted *R*^2^ = 0.637, *p* < 0.001, see Model 4 of Table 4). The results also revealed that states with a higher level of cultural tightness and college completion rate had a higher level of at least one dose of COVID-19 vaccination.

**Table 3:** Predictors of total population with at least one dose COVID-19 vaccination across U.S.

		***B***	***SE***	***Beta***	***t***	***p***
Model 3	1. Residential mobility	−168.162	69.1	−0.331	−2.434	0.019
	1. Residential mobility	−137.383	59.211	−0.271	−2.320	0.026
Model 4	2. Collectivism	−0.117	0.090	−0.139	−1.295	0.203
	3. Tightness	−0.310	0.103	−0.409	−2.998	0.005
	4. Case rate per 100 k	−0.001	0.001	−0.246	−1.611	0.115
	5. Death rate per 100 k	0.020	0.026	0.129	0.780	0.440
	6. Per-capita income	0.000	0.000	−0.163	−0.907	0.370
	7. Poverty rate (percent)	−0.287	0.693	−0.080	−0.414	0.681
	8. Completing college (percent)	0.730	0.323	0.401	2.265	0.029
	9. Real GDP in chained dollars	−6.658	0.000	−0.034	−0.338	0.737

**Table 4:** Fitness of regression models on residential mobility and COVID-19 vaccination rates.

	***R***	***R^2^***	**Adjusted *R^2^***	***F***	***p***
Model 1	0.384	0.147	0.129	8.278	0.006
Model 2	0.924	0.854	0.821	25.927	0.000
Model 3	0.331	0.110	0.091	5.922	0.019
Model 4	0.839	0.704	0.637	10.565	0.000

### Study 2

**Figure 1:**
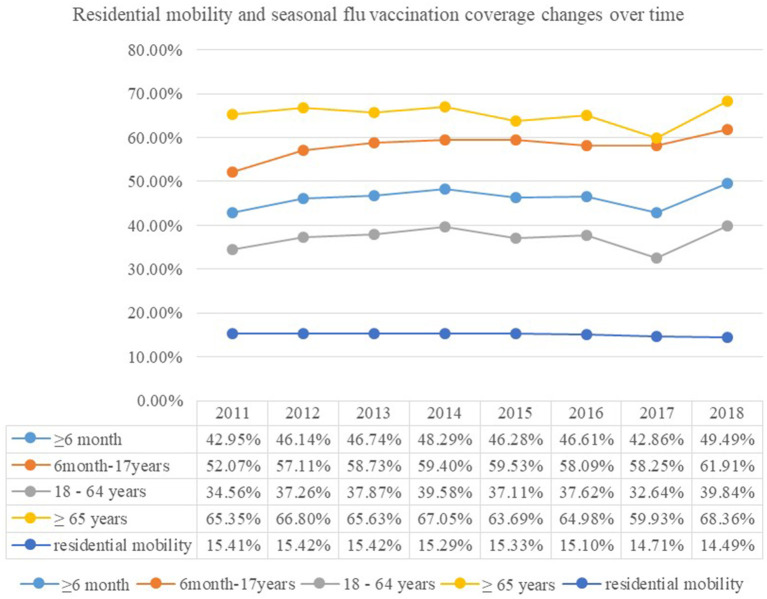
Residential mobility and seasonal flu vaccination coverage changes over time.

The average flu vaccination rate of population over 6 months is 46.2%. The population over 65 years old showed the highest vaccination rate of 65.2% (see Table 5). Residential mobility is negatively related to state-level collectivism (*r* = −0.331, *p* < 0.001), real per-capita personal income (*r* = −0.154, *p* < 0.001), real GDP in chained dollars (*r* = −0.218, *p* < 0.001), and seasonal flu vaccination coverage (*r* = −0.345, *p* < 0.001) of all age groups (see Table 5). The changes in residential mobility and seasonal flu vaccination coverage over time are presented in Figure 1.

**Table 5:** Descriptive statistics of main variables of study 3.

	***M***	***SD***	**1**	**2**	**3**	**4**	**5**	**6**	**7**	**8**
1. Residential mobility	0.151	0.022								
2. Real per capita personal income	46,788.607	5,411.051	−0.154^**^							
3. Real GDP in chained dollars	333,670.767	415,811.724	−0.218^**^	0.075						
4. Collectivism	49.997	11.237	−0.331^**^	−0.359^**^	0.216^**^					
5. Tightness	50.151	12.384	0.038	−0.217^**^	−0.123^*^	0.230^**^				
6. ≥6 months vaccination coverage	0.462	0.051	−0.345^**^	0.333^**^	−0.087	−0.031	−0.065			
7.6 months−17 years vaccination coverage	0.581	0.078	−0.477^**^	0.387^**^	0.071	0.142^**^	−0.192^**^	0.798^**^		
8.17–64 years vaccination coverage	0.370	0.052	−0.219^**^	0.322^**^	−0.145^**^	−0.121^*^	−0.07	0.959^**^	0.643^**^	
9. ≥65 years vaccination coverage	0.652	0.057	−0.200^**^	0.062	−0.056	−0.009	0.157^**^	0.729^**^	0.332^**^	0.703^**^

* *p* < 0.05,

** *p* < 0.01.

### Regression analyses

Regression analyzes showed that residential mobility negatively predicts flu vaccination coverage estimate of all age groups in general (*B* = −0.789, *SE* = 0.117, *t* = −6.77, *p* < 0.001, 95% CI = [−1.018, −0.56], see Model 1 of Table 6), such that states with a higher level of residential mobility had a lower percentage of people receiving flu vaccination in the corresponding flu season. However, further subgroup analyzes indicated that the results differ across years and age groups. For example, residential mobility did not predict seasonal flu vaccination coverage of the population over 65 years in the years 2014, 2015, 2017, and 2018 and of the population 18–64 years in 2012, 2014, 2017, and 2018. But it is noteworthy that residential mobility constantly predicts seasonal flu vaccination coverage of the population 6 months−17 years from 2011 to 2018 flu season (see Table 6).

**Table 6:** Regression analyzes of seasonal flu vaccination rates among different age group.

**Year**	**Age groups**	***B***	***SE***	***Beta***	***t***	***p***	***Adjusted R^2^***	***F***	***p***
2011–2018 RM	≥6 M	−0.789	0.117	−0.338	−6.770	0.000	0.222	23.585	0.000
	6 M−17 Y	−1.297	0.161	−0.362	−8.063	0.000	0.368	47.023	0.000
	18 Y−64 Y	−0.564	0.122	−0.238	−4.621	0.000	0.169	17.153	0.000
	≥65 Y	−0.659	0.141	−0.254	−4.675	0.000	0.087	7.742	0.000
2011 RM	≥6 M	−0.733	0.249	−0.404	−2.949	0.005	0.266	4.553	0.002
	6 M−17 Y	−1.062	0.401	−0.357	−2.651	0.011	0.289	4.978	0.001
	18 Y−64 Y	−0.516	0.253	−0.290	−2.039	0.048	0.210	3.608	0.008
	≥65 Y	−0.818	0.338	−0.345	−2.421	0.020	0.207	3.552	0.009
2012 RM	≥6 M	−0.790	0.309	−0.356	−2.557	0.014	0.207	3.558	0.009
	6 M−17 Y	−1.380	0.454	−0.387	−3.038	0.004	0.335	5.944	0.000
	18 Y−64 Y	−0.527	0.324	−0.237	−1.627	0.111	0.130	2.460	0.047
	≥65 Y	−0.696	0.346	−0.303	−2.013	0.050	0.071	1.744	0.145
2013 RM	≥6 M	−0.972	0.312	−0.444	−3.112	0.003	0.237	3.922	0.005
	6 M−17 Y	−1.314	0.424	−0.420	−3.102	0.003	0.315	5.316	0.001
	18 Y−64 Y	−0.758	0.327	−0.341	−2.317	0.025	0.188	3.169	0.016
	≥65 Y	−0.971	0.334	−0.417	−2.902	0.006	0.228	3.783	0.006
2014 RM	≥6 M	−0.739	0.294	−0.358	−2.511	0.016	0.181	3.163	0.016
	6 M−17 Y	−1.163	0.421	−0.362	−2.762	0.008	0.310	5.409	0.001
	18 Y−64 Y	−0.558	0.296	−0.261	−1.881	0.067	0.224	3.835	0.006
	≥65 Y	−0.594	0.344	−0.264	−1.725	0.092	0.059	1.617	0.175
2015 RM	≥6 M	−0.848	0.296	−0.416	−2.864	0.006	0.177	3.102	0.017
	6 M−17 Y	−1.506	0.483	−0.416	−3.119	0.003	0.305	5.310	0.001
	18 Y−64 Y	−0.638	0.280	−0.337	−2.280	0.028	0.146	2.671	0.034
	≥65 Y	−0.554	0.382	−0.234	−1.450	0.154	−0.015	0.852	0.521
2016 RM	≥6 M	−1.116	0.333	−0.487	−3.348	0.002	0.267	4.571	0.002
	6 M−17 Y	−1.792	0.448	−0.513	−4.001	0.000	0.431	8.420	0.000
	18 Y−64 Y	−0.796	0.339	−0.354	−2.349	0.023	0.216	3.704	0.007
	≥65 Y	−0.993	0.406	−0.410	−2.446	0.019	0.029	1.290	0.285
2017 RM	≥6 M	−0.715	0.312	−0.340	−2.289	0.027	0.178	3.127	0.017
	6 M−17 Y	−1.554	0.471	−0.421	−3.297	0.002	0.394	7.366	0.000
	18 Y−64 Y	−0.481	0.326	−0.224	−1.475	0.147	0.143	2.642	0.036
	≥65 Y	−0.221	0.508	−0.074	−0.436	0.665	−0.077	0.297	0.912
2018 RM	≥6 M	−0.679	0.364	−0.269	−1.866	0.069	0.184	3.167	0.016
	6 M−17 Y	−1.117	0.510	−0.299	−2.191	0.034	0.268	4.512	0.002
	18 Y−64 Y	−0.563	0.396	−0.208	−1.423	0.162	0.161	2.836	0.027
	≥65 Y	−0.468	0.301	−0.242	−1.557	0.127	0.050	1.502	0.209

Controlled variables of the regression models were collectivism, tightness, real per capita personal income and real GDP in chained dollars; RM, Residential Mobility; M, month; Y, years.

## Discussion

Study 1 demonstrated that residential mobility predicted lower levels of COVID-19 vaccination rates across the United States after controlling for a broad set of cultural and sociodemographic variables. The results that state-level residential mobility predicted vaccination rates for COVID-19 within a single, large country indicated the robustness of the relationship between residential mobility and vaccination behaviors during an emergent major public health crisis during the 21^st^ century. Study 2 provided further evidence that state-level residential mobility negatively predicted seasonal flu vaccination rate in each state after controlling for relevant sociocultural variables though the effect was inconsistent for those over 65 years old and 18–64 years old in some of the flu seasons. The results that residential mobility reduced overall seasonal flu vaccination rates across 8 years corroborated the negative impact of residential mobility on vaccination behaviors for seasonal flu.

Although residential mobility is primarily driven by human's pursuit of better education, career, living, and happiness (2), research also revealed the downside consequences of residential mobility such as a lower willingness to engage in procommunity behaviors (24). The current research provided further evidence on the negative effects of residential mobility, such that it could lead to lower vaccination rates for pandemics of infectious diseases. Given that the COVID-19 pandemic posed a social dilemma for balancing personal needs and collective safety (23) and that residential mobility could increase the primacy of personal self (2), a higher level of residential mobility might induce decisions that benefit the personal self but compromise collective welfare (e.g., low vaccination rates).

The current research contributes to the research on residential mobility by demonstrating its negative impact on vaccination behaviors during pandemics of infectious diseases. The results that residential mobility negatively predicts vaccination rates for COVID-19 (Study 1) and seasonal flu from 2011–2012 to 2018–2019 flu season (Study 2) after controlling for relevant variables such as collectivism, cultural tightness vs. looseness, and sociodemographic variables across 50 US states corroborate the downside of residential mobility on vaccination behavior. From a socioecological perspective, this research enriches our understanding of antecedents of vaccination behavior by documenting that a high level of residential mobility might be a societal barrier to promoting vaccination behavior. Socioecological factors (e.g., residential mobility) should be taken into account when designing specific and personalized strategies to promote vaccination for infectious diseases. Future research is warranted to further investigate the underlying mechanisms that account for the impacts of residential mobility on vaccination behaviors. For example, whether residential mobility reduces people's identification with their community or country, and in turn, leads to a lower level of vaccination behaviors. A better understanding of the underlying mechanisms will have implications for developing effective and scalable interventions to promote vaccination behavior during pandemics of infectious diseases.

### Limitations and future directions

The current research has several limitations that need to be addressed in future research. First, although the current research found that residential mobility predicted vaccination behaviors, the results are correlational rather than causal, and future research is needed to verify the causal relationship between residential mobility and vaccination behaviors. Research on the causal effect of residential mobility on vaccination behaviors would increase the internal validity of our research results. However, our results based on real-world data on vaccination rates for COVID-19 and seasonal flu enhanced the external validity of the relationship between residential mobility and vaccination behaviors. Second, the current research found that residential mobility predicted vaccination behaviors across the United States, and future research is warranted to investigate how cross-country variations in residential mobility predicted vaccination behaviors across different countries though the fact that regional or individual differences in residential mobility predicted vaccination behaviors are a robust test of the influence of residential mobility as it controlled other relevant country-level factors that could influence vaccination behaviors. Third, the data for vaccination rates of COVID-19 in Study 1 were accessed on 15 July 2021, and the results might not be generalized for vaccination rates afterward. As the COVID-19 pandemic is still spreading among many countries, it is necessary to continue evaluating the impact of residential mobility on vaccination rates during COVID-19. Fourth, the current research investigated the impact of residential mobility on vaccination behaviors for both emergent (COVID-19) and regular (seasonal flu) pandemics of infectious diseases, and it is not clear whether the results could be generalized to vaccination behaviors for other diseases. Future research is warranted to further investigate how residential mobility influences vaccination behavior for other infectious diseases.

## Conclusion

Across two studies, we found that residential mobility reduced vaccination rates for COVID-19 (Study 1) and seasonal flu between the 2011–2012 and 2018–2019 flu seasons (Study 2) across the United States. This research highlights residential mobility as a socioecological barrier to increasing vaccination in the efforts to contain pandemics of infectious diseases such as COVID-19 and seasonal flu. As seasonal flu is a yearly pandemic and the COVID-19 pandemic will likely continue to exist for a long time, posing a substantial risk for the most vulnerable groups, future research in designing behavioral intervention strategies to promote vaccination needs to be aware of the negative impacts of residential mobility and take precautionary strategies accordingly.

## Data availability statement

The original contributions presented in the study are included in the article/supplementary material, further inquiries can be directed to the corresponding author.

## Ethics statement

Ethical review and approval was not required for the study on human participants in accordance with the local legislation and institutional requirements. Written informed consent from the participants was not required to participate in this study in accordance with the national legislation and the institutional requirements.

## Author contributions

NZ: conceptualization, funding acquisition, methodology, supervision, and writing the original draft. TJ: data curation, writing, reviewing, and editing. YZ: data curation and writing the methods and results sections. GZ: supervision, writing, reviewing, and editing. All authors contributed to the article and approved the submitted version.

## References

[1] Choi H Oishi S. The psychology of residential mobility: A decade of progress. Curr Opin Psychol. (2020) 32:72–5. 10.1016/j.copsyc.2019.07.008 31401423

[2] Oishi S. The psychology of residential mobility: Implications for the self, social relationships, and well-being. Perspect Psychol Sci. (2010) 5:5–21. 10.1177/1745691609356781 26162059

[3] Wang P,. (2021). Key characteristics of the migrant population in china. Available online at: https://news.cssn.cn/zx/bwyc/202108/t20210804_5351758.shtml (accessed August 31, 2021).

[4] Frost R,. (2020). Who is moving and why? Seven questions about residential mobility. Available online at: https://www.jchs.harvard.edu/blog/who-is-moving-and-why-seven-questions-about-residential-mobility (accessed August 31, 2021).

[5] International Organization for Migration. (2021). Data and research. Available online at: https://www.iom.int/data-and-research (accessed August 31, 2021).

[6] Nouvellet P Bhatia S Cori A Ainslie KEC Baguelin M Bhatt S. Reduction in mobility and covid-19 transmission. Nat Commun. (2021) 12:1090. 10.1038/s41467-021-21358-2 33597546 PMC7889876

[7] Yuki M Schug J. Psychological consequences of relational mobility. Curr Opin Psychol. (2020) 32:129–32. 10.1016/j.copsyc.2019.07.029 31491705

[8] Salvador CE Berg MK Yu Q San Martin A Kitayama S. Relational mobility predicts faster spread of covid-19: A 39-country study. Psychol Sci. (2020) 31:1236–44. 10.1177/0956797620958118 32915703

[9] Harris JE. Mobility was a significant determinant of reported COVID-19 incidence during the Omicron Surge in the most populous U.S. Counties. BMC Infect Dis. (2022) 22:691. 10.1186/s12879-022-07666-y 35971063 PMC9376582

[10] Guo J Deng C Gu F. Vaccinations, mobility and covid-19 transmission. Int J Environ Res Public Health. (2021) 19:97. 10.3390/ijerph19010097 35010357 PMC8751025

[11] Michie S West R. Behavioural, environmental, social, and systems interventions against covid-19. Bmj. (2020) 370:m2982. 10.1136/bmj.m2982 32723732

[12] Michie S West R Rogers MB Bonell C Rubin GJ Amlôt R. Reducing SARS-CoV-2 transmission in the uk: A behavioural science approach to identifying options for increasing adherence to social distancing and shielding vulnerable people. Br J Health Psychol. (2020) 25:945–56. 10.1111/bjhp.12428 32428385 PMC7276722

[13] West R Michie S Rubin GJ Amlôt R. Applying principles of behaviour change to reduce SARS-CoV-2 transmission. Nat Hum Behav. (2020) 4:451–9. 10.1038/s41562-020-0887-9 32377018

[14] Zhang N. Behavioral insights for containing the covid-19 pandemic: Some practices in china. Behavioral Science & Policy. (2020). 10.1353/bsp.2020.0027

[15] Zhang N Kou Y. Implicit theories of health, consideration of future consequences, and engagement in health protective behaviors during the covid-19 pandemic in china. J Health Psychol. (2022) 27:1462–9. 10.1177/13591053211017191 33983050

[16] Berg MK Yu Q Salvador CE Melani I Kitayama S. Mandated bacillus calmette-guérin (bcg) vaccination predicts flattened curves for the spread of covid-19. Sci Adv. (2020) 6:eabc1463. 10.1126/sciadv.abc1463 32923613 PMC7457335

[17] de Gier B Andeweg S Joosten R Ter Schegget R Smorenburg N van de Kassteele J. Vaccine effectiveness against SARS-CoV-2 transmission and infections among household and other close contacts of confirmed cases, the netherlands, february to may 2021. Euro Surveill. (2021) 26:2100640. 10.2807/1560-7917.ES.2021.26.31.2100640 34355689 PMC8343550

[18] Greenwood B. The contribution of vaccination to global health: Past, present and future. Philos Trans R Soc Lond B Biol Sci. (2014) 369:20130433. 10.1098/rstb.2013.0433 24821919 PMC4024226

[19] Guiso N. Impact of vaccination on the infectious diseases epidemiology: Example of pertussis. Med Sci (Paris). (2007) 23:399–403. 10.1051/medsci/2007234399 17433230

[20] MacDonald NE. Vaccine hesitancy: Definition, scope and determinants. Vaccine. (2015) 33:4161–4. 10.1016/j.vaccine.2015.04.036 25896383

[21] Machingaidze S Wiysonge CS. Understanding covid-19 vaccine hesitancy. Nat Med. (2021) 27:1338–9. 10.1038/s41591-021-01459-7 34272500

[22] Wakefield JRH Khauser A. Doing it for us: Community identification predicts willingness to receive a covid-19 vaccination via perceived sense of duty to the community. J Community Appl Soc Psychol. (2021) 31:603–14. 10.1002/casp.2542 34220178 PMC8239513

[23] Van Lange PAM Rand DG. Human cooperation and the crises of climate change, covid-19, and misinformation. Annu Rev Psychol. (2022) 73:379–402. 10.1146/annurev-psych-020821-110044 34339612

[24] Oishi S Rothman AJ Snyder M Su J Zehm K Hertel AW. The socioecological model of procommunity action: The benefits of residential stability. J Pers Soc Psychol. (2007) 93:831–44. 10.1037/0022-3514.93.5.831 17983303

[25] Oishi S Ishii K Lun J. Residential mobility and conditionality of group identification. J Exp Soc Psychol. (2009) 45:913–9. 10.1016/j.jesp.2009.04.028

[26] Lu JG Jin P English AS. Collectivism predicts mask use during covid-19. Proc Natl Acad Sci U S A. (2021) 118:e2021793118. 10.1073/pnas.2021793118 34016707 PMC8201834

[27] Habersaat KB Betsch C Danchin M Sunstein CR Böhm R Falk A. Ten considerations for effectively managing the covid-19 transition. Nat Hum Behav. (2020) 4:677–87. 10.1038/s41562-020-0906-x 32581299

[28] Bavel JJV Baicker K Boggio PS Capraro V Cichocka A Cikara M. Using social and behavioural science to support covid-19 pandemic response. Nat Hum Behav. (2020) 4:460–71. 10.1038/s41562-020-0884-z 32355299

[29] Zhang N Yang S Jia P. Cultivating resilience during the covid-19 pandemic: A socioecological perspective. Annu Rev Psychol. (2022) 73:575–98. 10.1146/annurev-psych-030221-031857 34579547

[30] [Dataset] U,.S. Census Bureau. American community survey. (2021). Available online at: https://www.census.gov/programs-surveys/acs/ (accessed July 15, 2021).

[31] [Dataset] U,.S. COVID Data Tracker (CDC). Percent of total population fully vaccinated by state of residence percent of total population with at least one dose by state of residence. (2021). Available online at: https://covid.cdc.gov/covid-data-tracker/#datatracker-home (accessed July 15, 2021).

[32] Harrington JR Gelfand MJ. Tightness-looseness across the 50 united states. Proc Natl Acad Sci U S A. (2014) 111:7990–5. 10.1073/pnas.1317937111 24843116 PMC4050535

[33] Wang Q Yang L Jin H Lin L. Vaccination against covid-19: A systematic review and meta-analysis of acceptability and its predictors. Prevent Med. (2021) 150:106694. 10.1016/j.ypmed.2021.106694 34171345 PMC8217737

[34] Li L Wang J Nicholas S Maitland E Leng A Liu R. The intention to receive the covid-19 vaccine in china: Insights from protection motivation theory. Vaccines (Basel). (2021) 9:445. 10.3390/vaccines9050445 34063281 PMC8147465

[35] [Dataset] U,.S. Economic Research Service (ERS). The social economic demographic data. (2021). Available online at: https://www.ers.usda.gov.data-products/state-fac-sheets (accessed July 15, 2021).

[36] [Dataset] U,.S. Bureau of Economic Analysis (BEA). Real gdp in chained dollars of each state in 2019. (2021). Available online at: https://www.bea.gov/data/gdp (accessed July 15, 2021).

[37] [Dataset] U,.S. Centers for Disease Control Prevention (CDC). Seasonal influenza vaccination coverage. (2021). Available online at: https://covid.cdc.gov/covid-data-tracker/#datatracker-home (accessed July 15, 2021).

[38] Vandello JA Cohen D. Patterns of individualism and collectivism across the us. J Person Soc Psychol. (1999) 77:279–92. 10.1037/0022-3514.77.2.279

[39] [Dataset] U,.S. Bureau of Economic Analysis (BEA). Real per capita personal income (chained 2012 dollars) from 2011 to 2018. (2021). Available online at: https://www.bea.gov/data/income-saving (accessed August 16, 2021).

[40] [Dataset] U,.S. Bureau of Economic Analysis (BEA). Real gdp in chained dollars from 2011 to 2018. (2021). Available online at: https://www.bea.gov/data/gdp (accessed August 16, 2021).

[41] IBM Corp. Released (2020). IBM SPSS Statistics for Windows, Version 27.0. Armonk, NY: IBM Corp.

